# Nickel Sulfides Decorated SiC Foam for the Low Temperature Conversion of H_2_S into Elemental Sulfur

**DOI:** 10.3390/molecules23071528

**Published:** 2018-06-25

**Authors:** Cuong Duong-Viet, Lam Nguyen-Dinh, Yuefeng Liu, Giulia Tuci, Giuliano Giambastiani, Cuong Pham-Huu

**Affiliations:** 1 Institute of Chemistry and Processes for Energy, Environment and Health (ICPEES), UMR 7515 CNRS-University of Strasbourg (UdS), 25, rue Becquerel, 67087 Strasbourg CEDEX 02, France; 2 Ha-Noi University of Mining and Geology, 18 Pho Vien, Duc Thang, Bac Tu Liem, Ha-Noi, Vietnam; 3 The University of Da-Nang, University of Science and Technology, 54, Nguyen Luong Bang, Da-Nang, Vietnam; ndlam@dut.udn.vn; 4 Dalian National Laboratory for Clean Energy (DNL), Dalian Institute of Chemical Physics, Chinese Academy of Science, 457 Zhongshan Road, Dalian 116023, China; 5 Institute of Chemistry of OrganoMetallic Compounds, ICCOM-CNR Via Madonna del Piano, Sesto F.no, 10-50019 Florence, Italy; giulia.tuci@iccom.cnr.it; 6 Kazan Federal University, 420008 Kazan, Russia

**Keywords:** silicon carbide, H_2_S oxidation, low-temperature, catalysis, nickel sulfide

## Abstract

The selective oxidation of H_2_S to elemental sulfur was carried out on a NiS_2_/SiC^foam^ catalyst under reaction temperatures between 40 and 80 °C using highly H_2_S enriched effluents (from 0.5 to 1 vol.%). The amphiphilic properties of SiC foam provide an ideal support for the anchoring and growth of a NiS_2_ active phase. The NiS_2_/SiC composite was employed for the desulfurization of highly H_2_S-rich effluents under discontinuous mode with almost complete H_2_S conversion (nearly 100% for 0.5 and 1 vol.% of H_2_S) and sulfur selectivity (from 99.6 to 96.0% at 40 and 80 °C, respectively), together with an unprecedented sulfur-storage capacity. Solid sulfur was produced in large aggregates at the outer catalyst surface and relatively high H_2_S conversion was maintained until sulfur deposits reached 140 wt.% of the starting catalyst weight. Notably, the spent NiS_2_/SiC^foam^ catalyst fully recovered its pristine performance (H_2_S conversion, selectivity and sulfur-storage capacity) upon regeneration at 320 °C under He, and thus, it is destined to become a benchmark desulfurization system for operating in discontinuous mode.

## 1. Introduction

Hydrogen sulfide (H_2_S), which is contained in industrial effluent gas emissions, is one of the most hazardous elements with a very high environmental impact due to its toxicity [1], strong odor and corrosive effects [2,3]. Nowadays, H_2_S is selectively broken down from industrial emissions through selective oxidation protocols operating at various reaction temperatures or liquid-phase adsorption processes [4,5,6,7]. Most of the catalytic oxidation processes for the H_2_S conversion to elemental sulfur are carried out in either discontinuous- or continuous-mode. While in the former mode (typically operating at T < 180 °C) sulfur is trapped inside the catalyst and its regeneration (sulfur melting/vaporization in an inert atmosphere at temperatures around 350 °C) is periodically required; in the latter (T > 180 °C), sulfur is continuously removed from the catalyst and accumulated in the solid state at the reactor outlet. For these processes, the sulfur yield commonly ranges between 85–95% and it can be increased by adding an absorption stage at the outlet of the catalytic reactor. For low-temperatures discontinuous-modes, the rapid catalyst pore saturation by sulfur deposits limits the process to effluent gases containing low H_2_S concentrations only. Previous results obtained by some of us [8,9] showed that NiS_2_ supported on silicon carbide (SiC) grains (typical SiC size between 0.25–1 mm) catalyzes the selective H_2_S oxidation to elemental sulfur under mild conditions (i.e., 60 °C) and for H_2_S concentrations ≤ 0.2 vol.%. In terms of the catalyst’s efficiency, H_2_S reduction generates up to a maximum of 60 wt.% of sulfur deposits (with respect to the starting catalyst weight) before the spent system undergoes regeneration. Trovarelli et al. [10] have reported selective H_2_S oxidation at room temperature on activated charcoal. Their catalytic system displays markedly high desulfurization performance with complete H_2_S conversion into elemental sulfur. However, deactivation occurs rapidly in their system due to the progressive clogging of the catalyst micropores caused by formed sulfur deposits that hamper regular H_2_S uptake to the material active sites. In addition, the catalyst thermal treatment for the sulfur deposits removal does not fully restore the original performance of the material due to irreversible sulfur trapped in the catalyst micropores. Sun et al. [11] have reported on the use of nitrogen-rich mesoporous carbons as efficient single-phase catalysts for the selective oxidation of H_2_S (1000 ppmv; 0.1 vol.%) at conditions near to room temperature. Despite its high desulfurization performance, the powdery texture of their catalytic system limits its practical application. Indeed, powders have severe technical and logistic drawbacks, particularly for their application and use in industrial plants: powdery catalysts are less than easy to handle and transport and they can be responsible for high pressure drops across the catalyst bed under operational conditions. In addition, the moderate H_2_S concentrations allowed with these materials (i.e., 0.1 vol.%), together with the costly and energy consuming procedures applied to their synthesis, largely limit their industrial exploitation.

The quest for new catalytic materials with high selectivity and efficiency for H_2_S desulfurization, which are able to reduce the impact of sulfur deposits on their performance, and that have the ability to operate under low-temperature conditions in a discontinuous mode and preferably in the presence of relatively high H_2_S concentrations in the gaseous effluent, is a challenging priority for those engaged in the area of catalysis research. 

Highly porous macroscopic materials with controlled surface area and pore-size distribution are ideal supports for the catalytically active phases that are used in the treatment of various H_2_S containing effluents, including those characterized by high H_2_S concentrations (i.e., ≥0.5%). In addition, easy processing and handling of macroscopic matrices along with their thermal stability and shape adaptability are key requisites for the preparation of effective catalytic systems to be employed for H_2_S treatment in industrial plants.

Solid ceramic foams as catalyst supports were initially investigated by Twigg and Richardson [12] as substituents of honeycomb straight channel ceramic monoliths. Compared to the latter supports, foams display an extremely higher degree of radial mixing of the flow (gaseous or liquid) passing through their porous network, which improves the mixing of the reactants and heat transfer phenomena (in the case of thermally conductive supports). Solid foams and monoliths employed as catalytic stirrers have also been widely investigated by Schouten and co-workers [13,14,15,16] for several gas- and liquid-phase reactions. Among various foam supports, silicon carbides (SiCs) with their high open porosity, relatively large surface areas and medium thermal conductivity have found application as inert supports in the development of catalysts for a number of relevant catalytic transformations [17,18,19,20,21,22,23,24]. Their open cell structure reduces the pressure drop phenomena through the catalyst bed [25,26,27], while gas effluent turbulences originating within the foam axes [28,29] maximize contact between reactants and the catalyst active phase [30,31,32].

In this paper we describe the preparation of a nickel sulfide-decorated SiC foam (NiS_2_/SiC^foam^) to be employed as a highly efficient catalyst for the H_2_S oxidation to elemental sulfur under discontinuous mode. At odds with SiC-grains or SiC-extrudates as supports, the open cell structure of the foam composite provides a highly effective catalyst with a high retention capacity for solid sulfur deposits (over 140 wt.%) and reduced pore clogging effects, ideal for the treatment of gaseous effluents containing relatively high H_2_S concentrations. The presence of a passivated thin layer of SiO_2_/SiO_x_C_y_ at the SiC surface (formed during the final calcination of the support at 800 °C in air) gives the support a dual hydrophilic/hydrophobic character, which plays a key role in the anchoring and stabilization of the metal active phase [33] as well as in the sulfur storage mechanism throughout the low temperature desulfurization process. Finally, the thermal conductivity of SiC limits the formation of “hot spots” [34], thus avoiding the occurrence of side-reactions, which are particularly detrimental to the process selectivity (i.e., H_2_S over-oxidation). 

In this study, desulfurization runs were conducted in a discontinuous mode within the 40–80 °C temperature range and at relatively high H_2_S concentrations in the effluent (from 0.5 up to 1 vol.%). NiS_2_/SiC^foam^ showed almost quantitative H_2_S conversions, extremely high sulfur selectivity (from 96% to 99.6% at 80 and 40 °C, respectively) and unprecedented sulfur retention capacity (>140 wt.%), working at a gas hourly space velocity (GHSV) close to that employed in industrial plants. 

## 2. Experimental Section

### 2.1. General Procedure for the Preparation of NiS_2_-Decorated SiC; Materials and Methods

β-SiC supports (cubic foams (4 × 4 × 4 mm; *h* × *w* × *d*), ~0.06 cm^3^, 20 pores per inch (PPI); extrudates (3 × 1 mm; *h* × Ø), ~0.002 cm^3^ and rings (2 × 3 × 2 mm; *h* × Ø_ext_ × Ø_int_), ~0.008 cm^3^) were provided by SICAT SARL (www.sicatcatalyst.com). NiS_2_ decorated SiC samples were prepared via an incipient wetness impregnation method with a 5 wt.% Ni(NO_3_)_2_·H_2_O aqueous solution, following a slightly modified protocol [8]. In brief, the impregnated SiC solid was oven-dried at 110 °C overnight followed by calcination at 300 °C in air to convert the nickel salt into its corresponding nickel oxide. Afterwards, the treatment of NiO/SiC samples at 300 °C with a mixture of H_2_S (4 vol.%)/He (50 mL/min) for 2 h [35] leads to the corresponding NiS_2_/SiC composites.

### 2.2. Selective Oxidation of H_2_S to Elemental Sulfur

The main H_2_S oxidation processes are outlined below in Equations (1)–(3). In the present study, the highly selective H_2_S oxidation to elemental sulfur (Equation (1)) was carried out on NiS_2_/SiC composites as catalysts in a glass tubular reactor working isothermally at atmospheric pressure. A schematic representation of the desulfurization apparatus is provided in Figure S1 of the Supplementary Material.

H_2_S + ½O_2_   1/nS_n_ + H_2_O    ∆H = −222 kJ/mol (1)

S + O_2_   SO_2_         ∆H = −297 kJ/mol (2)

2H_2_S + 3O_2_   2SO_2_ + 2H_2_O   ∆H = −381 kJ/mol (3)

For the catalytic H_2_S oxidation, 3 g of the catalyst were charged on a silica wool in a tubular Pyrex reactor (inner diameter: 16 mm) located inside a vertical tubular electrical furnace. The temperature was controlled by a K-type thermocouple and a Minicor regulator. The gas mixture of reactants (H_2_S (0.5 and 1 vol.%), O_2_ (1.25 and 2.5 vol.%), H_2_O (30 vol.%) and He (balance) was passed downward through the catalyst bed. The gas flow rates were monitored by Brooks 5850TR mass flow controllers linked to a control unit. The gas hourly space velocity (GHSV) was fixed at 1200 h^−1^ and the O_2_/H_2_S molar ratio was constantly kept at 2.5. The steam (30 vol.%) in the reactant feed was provided by bubbling a He flow through a saturator containing hot water at 80 °C. The reaction was conducted in a discontinuous mode in the 40–80 °C temperature range. The sulfur formed in the reaction was steadily condensed inside the catalyst bed and periodically vaporized (thermal regeneration process of the spent catalyst under Helium at 320 °C for 2 h) and condensed at the reactor outlet in a trap maintained at room temperature. The analysis of the inlet and outlet gases was performed in real time using a Varian CP-3800 gas chromatograph (GC) equipped with a Chrompack CP-SilicaPLOT capillary column and a thermal catharometer detector (TCD) for the detection of O_2_, H_2_S, H_2_O and SO_2_. The TCD sensibility was previously calibrated and the limit detection level was fixed at 50 ppm for H_2_S and 40 ppm for SO_2_. Long term reaction tests were carried out to assess the stability of the catalytic materials in the process. Finally, blank desulfurization tests carried out on the plain SiC supports (without nickel) were used to demonstrate the inertness of the support in the process.

H_2_S conversion (X_H2S_), S selectivity (S_S_) and S yield (Y_S_) were calculated according to Equations (4)–(6):

(4) $$\;X_{H_{2}S}=\;\frac{[H_{2}S{]}_{\mathrm{in}}-[H_{2}S{]}_{\mathrm{out}}}{{\left[H_{2}S\right]}_{\mathrm{in}}}\;\cdot 100$$

(5) $$\;S_{S}=\;\left(1-\;\frac{{\left[{\mathrm{SO}}_{2}\right]}_{\mathrm{out}}}{[H_{2}S{]}_{\mathrm{in}}-\;[H_{2}S{]}_{\mathrm{out}}}\right)\cdot 100$$

(6) $$\;Y_{S}={\;X}_{H_{2}S}\cdot S_{S}$$

### 2.3. Characterization Techniques

Scanning electron microscopy (SEM) (ZEISS, Oberkochen, Germany) was carried out on a ZEISS GeminiSEM 500 microscope with a resolution of 5 nm. The samples were deposited onto a double face graphite tape in order to avoid charging effect during the analysis. Elemental analyses were conducted on an inductive coupled plasma mass spectrometry (ICP-MS). A powder X-ray diffraction study was run on a Bruker D-8 Advance (Bruker Ins., Wissembourg, France) with a CoKα radiation in a θ/2θ mode. The nature of the crystalline phase in the sample was indexed using the data base of the Joint Committee on Powder Diffraction Standards (JCPDS). The average particle size was calculated according to the Scherrer equation (B (2θ) = Kλ/Lcos θ, where B is a Bräggs angle (2θ), L is the crystal size and λ is the X-ray radiation)). Specific surface areas (SSA) and pore volume distributions were determined on a Micromeritics sorptometer (Micromeritics, Norcross, USA) using N_2_ as adsorbent at the liquid N_2_ temperature. All samples were previously outgassed at 250 °C under vacuum for 8 h in order to desorb moisture and adsorb species from their surface. The X-ray photoelectron spectroscopy (XPS) measurements of the support and catalyst were performed by using a MULTILAB 2000 (THERMO) spectrometer (Thermo Fisher Scientific, Waltham, MA, USA) equipped with an AlKα anode (hν = 1486.6 eV) with 10 min of acquisition to achieve a good signal to noise ratio. Peak deconvolution was performed with the “Avantage” program from the Thermoelectron Company. The C1s photoelectron binding energy was set at 284.6 ± 0.2 eV relative to the Fermi level and used as reference to calibrate the other peak positions. The transmission electron microscopy (TEM) analysis was performed on a JEOL 2100F instrument (JEOL, Tokyo, Japan) working at a 200 kV accelerated voltage, equipped with a probe corrector for spherical aberrations, and with a point-to-point resolution of 0.2 nm. The sample was ground and dispersed by ultrasound in an acetone solution for 5 min and then a drop of the solution was deposited on a copper grid covered with a holey carbon membrane for observation.

## 3. Results and Discussion

### 3.1. NiS_2_/SiC Synthesis and Characterization

NiS_2_/SiC catalysts were prepared according to procedures in the literature [8,9] using macroscopically shaped β-SiC supports. In a typical procedure, the selected SiC matrix was impregnated with a 5 wt.% aqueous solution of nickel nitrate before undergoing calcination (see Experimental Section). Then, NiO nanoparticles were converted into NiS_2_ via sulfidation reaction under a H_2_S/He flow [35]. In the present study, the most effective β-SiC support selected for the preparation of the desulfurization catalyst consists of cubic foams (4 × 4 × 4 mm; *h* × *w* × *d*, ~0.06 cm^3^ as average dimension) featured by relatively large cell sizes (20 pores per inch (PPI)) and narrow strut thickness (0.1 ÷ 0.15 mm) for an appropriate reactants accessibility and low pressure drop. Two other β-SiC supports, extrudates (3 × 1 mm; *h* × Ø, ~0.002 cm^3^ as average dimension) and rings (2 × 3 × 2 mm; *h* × Ø_ext_ × Ø_int_, ~0.008 cm^3^ as average dimension) featuring a thicker equivalent strut (~1.0 mm) were selected for comparative studies.

**Figure 1:**
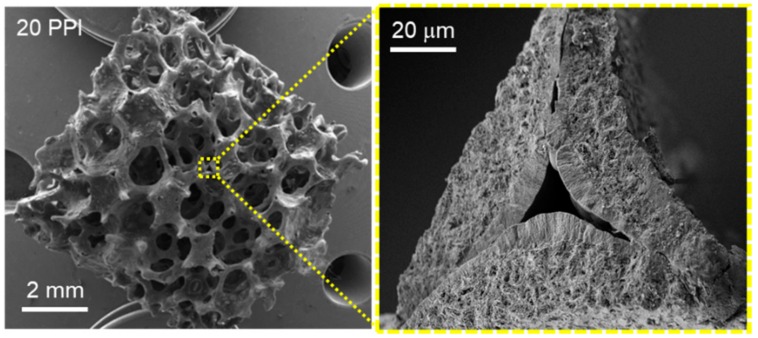
(**Left**) SEM micrograph of the SiC foam (20 PPI, ~0.06 cm^3^) and (**Right**) micrograph of a foam strut detail featuring with hollow and porous structure. The SiC foam materials were provided by SICAT SARL (www.sicatcatalyst.com).

SEM and HR SEM micrographs of SiC foam show an open cell structure featuring 3D interconnected channels and characterized by a relatively high material porosity along with sharp-cornered cavities (Figure 1).

**Figure 2:**
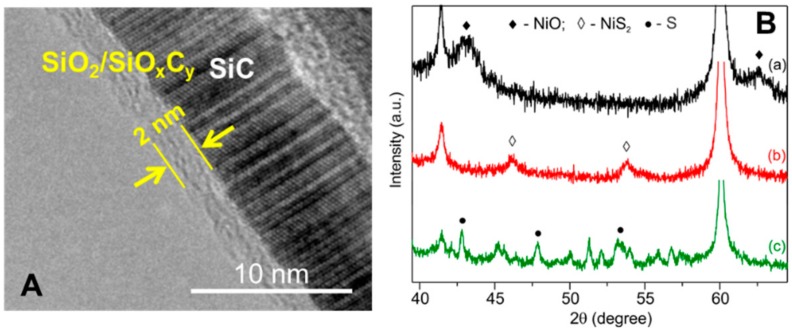
(**A**) TEM micrograph showing the presence of a thin SiO_2_/SiO_x_C_y_ passivated layer on the surface of SiC; (**B**) Comparison of PXRD analyses of different samples: (a) NiO/SiC^foam^ catalyst, (b) freshly prepared NiS_2_/SiC^foam^ catalyst after sulfidation reaction with a mixture of H_2_S (4 vol.%)/He at 300 °C for 2 h, and (c) spent NiS_2_/SiC^foam^ catalyst containing sulfur after ~60 h of reaction at 60 °C with 0.5 vol.% of H_2_S in the effluent.

These morphological properties present a relatively high accessible surface area to the support along with an ideal macro- and meso-porosity for good dispersion of NiS_2_ nanoparticles. TEM analysis confirms the presence of a thin (~2 nm) passivated layer of SiO_2_/SiO_x_C_y_ on the SiC support (Figure 2A). Such an amorphous layer ensures a better interaction with the metal salt during the impregnation/calcination phase and allows a more effective anchorage of the metal active nanoparticles [33]. The SiO_2_/SiO_x_C_y_ phase, whose composition (SiO_2_:SiO_x_C_y_ = 40:60 at.%—confirmed by XPS analysis is in good accord with the data in the literature [36,37,38], is generated during a SiC post-synthetic calcination step. It is known, that the latter confers a prevalently hydrophilic character to the inner pore surface, whereas the remaining part of the support, mostly based on naked SiC, has a prevalently hydrophobic character [39,40]. Accordingly, impregnation with an aqueous nickel nitrate solution and subsequent Ni NPs formation will mainly occur within the hydrophilic pores of the SiC matrix. The elemental analysis (EA) carried out on the NiS_2_/SiC^foam^ sample indicates a nickel loading of 4.6 ± 0.5 wt.%, relatively close to the theoretical value (5 wt.%). Figure 2B shows the XRD pattern of NiO/SiC and NiS_2_/SiC, the latter before and after desulfurization catalysis. From the comparative analysis of the three samples it can be inferred that: (1) the sulfidation step successfully converts NiO NPs into the NiS_2_ active phase; (2) the average particle size for NiS_2_, as deduced from XRD line broadening, according to the Scherrer equation, is centered at 8 ± 1 nm; and (3) the NiO conversion to NiS_2_ (sulfidation conditions: H_2_S 4 vol.%/He at 300 °C for 2 h—see Experimental section) seems to be accompanied by a certain degree of NP sintering as the diffraction lines of the latter are slightly narrower compared to those associated with oxide precursors (Figure 2B, a vs. b).

Finally, the specific surface area (SSA) measured for the pristine SiC foam, NiO/SiC and NiS_2_/SiC composites (Table S1) did not show any distinctive variation among the samples except for a clear decrease in the average pore size in both Ni-decorated materials (Table S1 and Figure S2 on the Supplementary Material). 

The same impregnation/sulfidation procedure was adopted for preparing two other NiS_2_-decorated SiC matrices (extrudates and rings). Elemental analyses and X-ray diffraction studies carried out on these two additional heterogeneous systems, confirm similar Ni loading (4.6 ± 0.5 wt.%) and average nanoparticle size (see Figure S3 for NiO NPs on different SiC supports, Supplementary Material). For a complete material characterization, NiO NPs were prepared following the same impregnation procedure on a powdery SiC support and the resulting composite was employed for TEM analysis and characterization of Ni NPs size and distribution before the sulfidation step. As Figure S4 shows, the as-prepared sample, presents a rather homogeneous dispersion of the NiO NPs with a size distribution centered between 5 and 8 nm. 

### 3.2. Desulfurization Process

#### 3.2.1. Influence of SiC Support on the Desulfurization Performance

**Figure 3:**
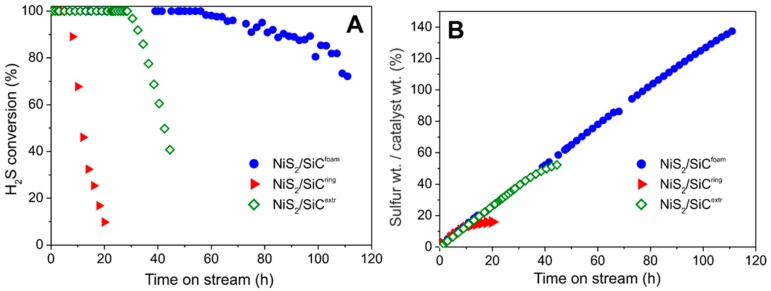
(**A**) H_2_S conversion on various NiS_2_/SiC^x^ catalysts (x = foam, extrudates, rings); (**B**) Solid sulfur deposited on the catalyst expressed as wt.% increase of the spent catalyst with respect to the initial catalyst weight. Reaction conditions: [H_2_S] = 0.5 vol.%, [O_2_] = 1.25 vol.%, O_2_-to-H_2_S ratio = 2.5, [H_2_O] = 30 vol.%, balance helium, reaction temperature = 60 °C, GHSV (STP) = 1200 h^−1^.

All NiS_2_-decorated SiC matrices were scrutinized as catalysts for the selective oxidation of highly H_2_S concentrated gas effluents under isothermal conditions (reactor temperature initially set at 60 °C). As Figure 3 shows, the NiS_2_/SiC^foam^ presents a markedly higher performance compared to the other NiS_2_-decorated SiC-matrices (rings and extrudates). The higher H_2_S conversion on NiS_2_/SiC^foam^ as a function of time-on-stream is mainly ascribed to the SiC foam strut thickness (0.1 ÷ 0.15 mm) and its highly mesoporous nature, which ensure an ideal diffusion of reactants towards the catalytically active sites. Indeed, the hollow, porous and interconnected network of SiC^foam^ ensures a higher catalyst stability on stream with respect to rings and extrudates supports, where already a small amount of sulfur deposits on the material surface prevents regular access of reactants to the Ni-active sites, mostly located inside pores. For streams with low H_2_S concentration, sulfur deposits are relatively small and they can be conveniently moved by films of condensed water (acting as a conveyor belt), from inner pores close to the metal active site (hydrophilic part of SiC), to adjacent areas (hydrophobic parts of SiC) where solid sulfur can be stored without inducing any pore clogging and thus catalyst deactivation. On the other hand, with higher H_2_S concentrations in the gas effluent, i.e., 0.5 vol.%, the large amounts of sulfur deposits formed can rapidly cause pore clogging if they are not efficiently dispersed through a fast internal transportation mechanism (vide infra). For these reasons, NiS_2_/SiC^foam^ is able to operate with higher H_2_S concentrations in the stream while ensuring an almost complete selectivity to the process (100% of elemental sulfur—vide infra) for long reaction times (Figure 3A), together with a physical sulfur-storage capacity largely exceeding the catalyst pristine weight (>140%; Figure 3B).

Indeed, when NiS_2_/SiC^ring^ and NiS_2_/SiC^extr^ are used as desulfurization catalysts under identical conditions, H_2_S conversion decreases after a few hours on stream with a deactivation rate of ca. 8%/h and 5%/h, respectively (Figure 3A). With NiS_2_/SiC^foam^, the desulfurization performance is markedly higher and deactivation takes place after many hours at a deactivation rate close to 0.5%/h. As a result, the amount of sulfur deposited on the three different spent samples (as a function of their time-on-stream) appears dramatically different. As Figure 3B shows, the wt./wt.% between deposited sulfur and initial catalyst weight, is close to 140% for NiS_2_/SiC^foam^ whereas it is around 50% and 20% for NiS_2_/SiC^extr^ and NiS_2_/SiC^ring^, respectively. Accordingly, NiS_2_/SiC^foam^ was the heterogeneous system of choice for the following desulfurization studies.

#### 3.2.2. Catalyst Regeneration and H_2_S Concentration Effects

**Figure 4:**
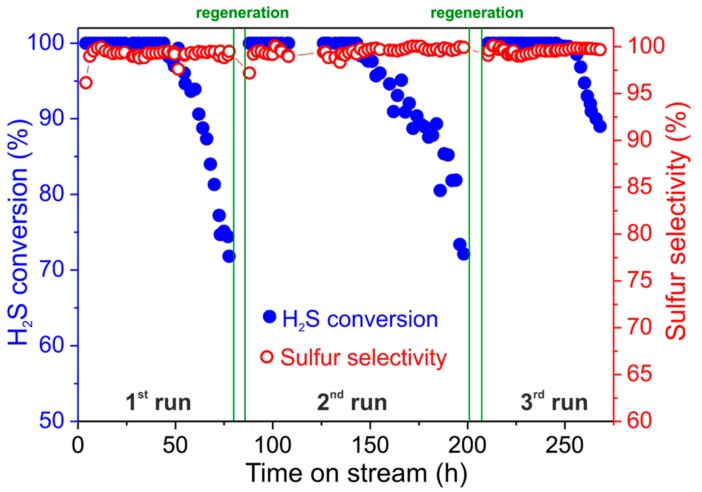
Desulfurization performance on NiS_2_/SiC^foam^ catalyst as a function of successive catalyst/regeneration cycles; blue spheres refer to H_2_S conversion and empty red spheres refer to sulfur selectivity. Reaction conditions: [H_2_S] = 0.5 vol.%, [O_2_] = 1.25 vol.%, O_2_-to-H_2_S ratio = 2.5, [H_2_O] = 30 vol.%, balance helium, reaction temperature = 60 °C, GHSV (STP) = 1200 h^−1^. Green lines indicate the catalyst regeneration time (320 °C in helium for 2 h).

For completing the desulfurization study with the NiS_2_/SiC^foam^ catalyst, the latter was produced from a macroscopic SiC foam cylinder (40 × 12 mm; *h* × Ø, ~4.5 cm^3^ as average dimension) and SiC powder (Ø of 0.08 mm) was used to fill up the empty spaces between the cylinder and the reactor walls in order to reduce the preferential diffusion path. At 60 °C with 0.5 vol.% of H_2_S in the gas effluent, H_2_S conversion is almost quantitative within fifty hours of the catalyst on stream. Under these conditions, sulfur selectivity is constantly over 99.5% even when catalyst deactivation starts due to the formation of sulfur deposits (Figure 4). After fifty hours the desulfurization performance gradually decreases with an H_2_S conversion close to 80% after 70 h on stream and a virtually unchanged sulfur selectivity (99.5%). The very high sulfur selectivity measured with this catalyst is mainly ascribed to the low operating temperature (60 °C) that limits the formation of sulfur over-oxidation by-products (namely SO_2_; see Equations (2) and (3)) [41,42].

In addition, the thermal conductivity of the SiC^foam^ support largely contributes to the high process selectivity in desulfurization of H_2_S enriched effluents. Indeed, the exothermic character of the reaction can be responsible for the generation of local “hot spots” in the catalyst that cause an undesired sulfur over-oxidation to SO_2_. The thermal conductivity of SiC^foam^ contributes to the rapid dissipation of the reaction heat (∆H = −222 kJ·mol^−1^), thus allowing a high process selectivity even for high H_2_S concentrations in the stream. Similar results have previously been discussed for other SiC^foam^-based catalysts in other exothermic processes such as Fischer-Tropsch synthesis (FTS) [43], dimethyl ether synthesis from methanol dehydration [44] and ZSM-5/SiC foam composites for methanol to propylene (MTP) application [45,46].

To the best of our knowledge, this result represents the first example of a long-term stable and selective desulfurization catalyst operating at low reaction temperature (60 °C) with relatively high H_2_S concentrations in the stream (0.5 vol.%) and exceptional sulfur retention capacity. Other state-of-the-art low temperature oxidation catalysts generally operate at H_2_S concentrations up to 0.2 vol.% maximum to avoid undesirable and rapid deactivation phenomena [8].

Catalyst regeneration was then performed by heating the exhaust catalyst at 320 °C under a He flow for 2 h. Afterwards, NiS_2_/SiC^foam^ can be reused under the same conditions in accordance with a discontinuous operating protocol (Figure 4). The reused catalyst shows desulfurization performances that are largely superimposable on those recorded for the fresh catalyst, thus indicating that regeneration takes place efficiently with complete sulfur removal and recovery of the catalyst’s active phase and morphology. Notably, the stability of the catalyst on stream after regeneration is even higher than that of the fresh sample. Indeed, the desulfurization reaction on the reused NiS_2_/SiC^foam^ increases its long-term stability (with quantitative H_2_S conversion) up to 65 h of the catalyst on stream. Such an improvement can be ascribed to an additional and beneficial conversion (sulfidation) of residual NiO NPs into the catalytically active phase (NiS_2_). Indeed, the regeneration step allows residual NiO NPs to be converted into NiS_2_ by the gaseous sulfur produced throughout the material thermal treatment in the He atmosphere. A similar behavior has already been described by some of us in a previous work on the topic [8]. Overall, NiS_2_/SiC^foam^ can be re-used with no appreciable decrease of its efficiency and selectivity even after several consecutive runs, thus confirming its applicability to desulfurization treatment of highly H_2_S enriched effluents under discontinuous mode. In addition, it represents a valuable alternative for replacing sulfur recovery units working at medium temperature (ca. 110 °C), i.e., Sulfreen and Oxo-Sulfreen processes [5], which are generally applied to the treatment of sour gases with low H_2_S concentrations (i.e., 0.5 to 4 vol.%), where the traditional combined Claus/Super-Claus process is not effective.

The generated sulfur is prevalently in the form of S_8_ with a cross-section of 1.5 nm^2^ per S_8_ molecule [47]. Accordingly, ~1 wt.% in sulfur (with respect to catalyst weight) would be enough to completely cover (monolayer) the catalyst surface, thus causing its definitive deactivation. On the other hand, we have demonstrated that sulfur deposits (in wt.) formed on the spent catalyst reach up to 140 wt.% of the starting catalyst weight and therefore they are more than those required for the generation of a passivating S_8_ monolayer coating. Therefore, S_8_ is not homogeneously dispersed on the catalyst surface but large sulfur deposits grow locally, thus ensuring the access of reagents to NiS_2_ NPs for the process to occur even after several hours on stream.

**Figure 5:**
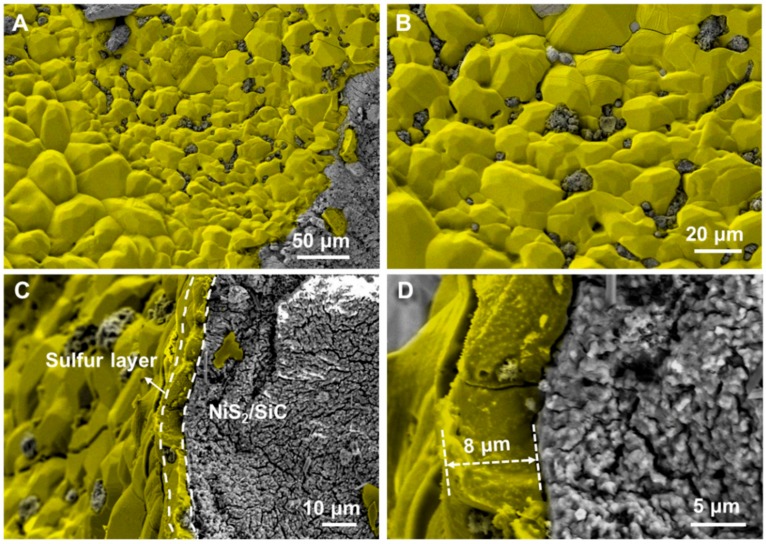
(**A**,**B**) SEM micrographs of the NiS_2_/SiC^foam^ surface after desulfurization test at 60 °C with 0.5 vol.% of H_2_S and 100 h catalyst on stream; pictures show the presence of large sulfur aggregates on the catalyst surface whereas a large part of the catalyst remains free of sulfur deposit. (**C**,**D**) Cross-section SEM micrographs showing the presence of a thick layer of sulfur on the catalyst surface. The solid sulfur is colored in yellow in all SEM micrographs.

SEM images of the spent NiS_2_/SiC^foam^ catalyst recorded at different magnifications, are shown in Figure 5. These micrographs reveal the presence of discrete sulfur patches covering part of the catalyst surface (Figure 5A,B) in the form of large aggregates with a cross-section of ca. 8 µm (Figure 5C,D), leaving part of the catalyst surface still accessible to the desulfurization process.

**Figure 6:**
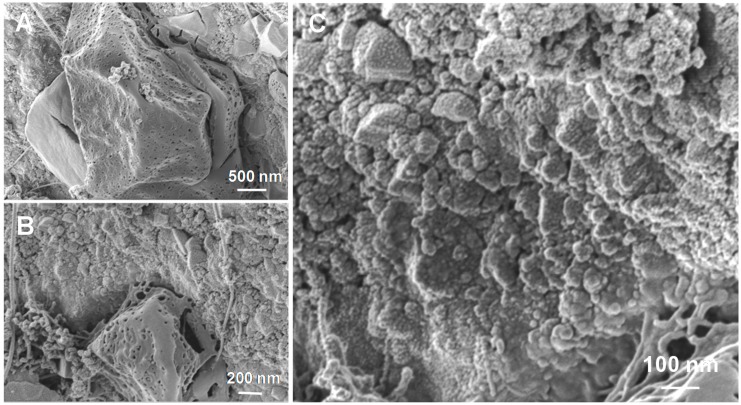
(**A**,**B**) High-resolution SEM micrographs of sulfur aggregates: from sulfur nanosized particles (i.e., 15 ± 4 nm, **C**) to larger sulfur aggregates featuring with numerous voids.

The high-resolution SEM micrographs in Figure 6A–C show the presence of large sulfur patches featuring a high density of voids within their structure, whose origin can be ascribed to the stepwise evolution of sulfur nanoparticles into larger aggregates during the course of the reaction. The SEM analysis also reveals the presence of small sulfur particles with variable sizes whose formation is ascribed to the aggregation of primary sulfur nuclei during the transportation and aggregation processes.

It is supposed that during the desulfurization process, nanoscopic sulfur deposits generated close to NiS_2_ are moved away from the active sites by the condensed steam (water film) to generate localized larger sulfur aggregates (mainly at the outer catalyst surface) or to enlarge the existing ones. This sulfur migration phenomenon is favored by the dual hydrophilic/hydrophobic character of the SiC support (i.e., prevalently hydrophilic in the inner pores and hydrophobic at their outer side) [33,48] that facilitates the sulfur agglomeration on the hydrophobic part of the catalytic material and keeps the hydrophilic pores accessible, where the active phase is (prevalently) located. This sulfur agglomeration path is also in accordance with our previous findings [8].

**Figure 7:**
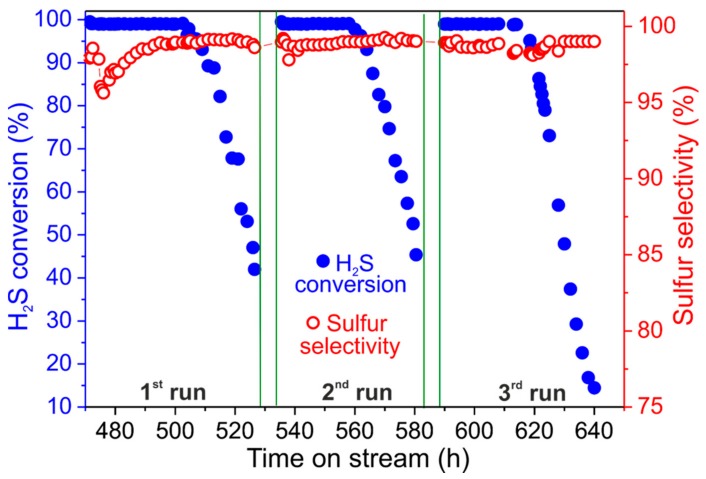
Desulfurization performance on NiS_2_/SiC^foam^ catalyst as a function of successive catalyst/regeneration cycles; blue spheres refer to H_2_S conversion and empty red spheres refer to sulfur selectivity. Reaction conditions: [H_2_S] = 1 vol.%, [O_2_] = 2.5 vol.%, O_2_-to-H_2_S ratio = 2.5, [H_2_O] = 30 vol.%, balance helium, reaction temperature = 60 °C, GHSV (STP) = 1200 h^−1^. Green lines indicate the catalyst regeneration time (320 °C in helium for 2 h).

For higher H_2_S concentrations in the stream (1 vol.%; O_2_-to-H_2_S = 2.5, 60 °C), H_2_S conversion and sulfur selectivity with NiS_2_/SiC^foam^ are slightly reduced to 99.6% and 99.4%, respectively (Figure 7). The slightly reduced H_2_S conversion and sulfur selectivity measured with NiS_2_/SiC^foam^ are ascribed to a local increase in the reactor temperature induced by the highly exothermic nature of the desulfurization process not sufficiently compensated by the thermal conductivity of the SiC support under these more severe desulfurization conditions (1 vol.% of H_2_S in the stream). Indeed, local hot spots inside the catalyst bed can be responsible for a partial NiS_2_ sintering with a subsequent reduction of the NPs active surface area (reduced H_2_S conversion) as well as a H_2_S over-oxidation with increased SO_2_ production (reduced sulfur selectivity).

The catalyst maintains its extremely high sulfur yield for about 33 h on stream, then a fast deactivation takes place as a consequence of the pore clogging of the catalyst caused by the formation of sulfur deposits. In spite of a faster catalyst deactivation rate (compared to 0.5 vol.% of H_2_S in the stream), the final wt./wt.% ratio between produced sulfur and pristine catalyst weight is maintained almost unchanged. Indeed, the total sulfur deposit on the spent catalyst is roughly 140 wt.% of the starting catalyst weight. Overall, this result also confirms the excellent performance of NiS_2_/SiC^foam^, which is the highest reported so far for a catalytic system operating under discontinuous mode at low temperatures and relatively high H_2_S concentrations.

As Figure 7 shows, the regenerated catalyst (320 °C under He for 2 h) recovers its pristine performance (in terms of H_2_S conversion and sulfur selectivity) although deactivation occurs after 27 h on stream. As a matter of fact, on the second run the wt./wt.% measured between deposited sulfur on the spent catalyst and pristine catalyst weight is reduced from 140% to 110% and it is constantly maintained at the latter value for all successive catalytic trials under identical conditions. This faster catalyst deactivation on the reused NiS_2_/SiC^foam^ catalyst is also related to the modification of its active phase (reduced active surface area of sintered NiS_2_ NPs) as a consequence of the harsher desulfurization conditions used (1 vol.% of H_2_S in the stream).

As a proof of this concept, NiS_2_/SiC^foam^ maintains stable desulfurization performance for all runs successive to the first catalytic/regeneration cycle, thus indicating that no additional NiS_2_ sintering occurs.

#### 3.2.3. Influence of the Reaction Temperature

**Figure 8:**
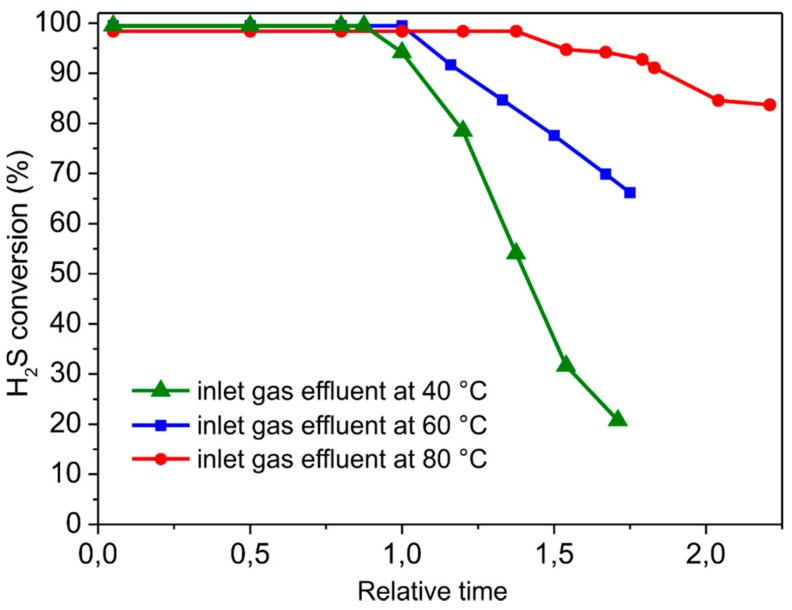
Influence of the reaction temperature on H_2_S conversion as a function of the relative breakthrough time with NiS_2_/SiC^foam^ catalyst. The breakthrough time for NiS_2_/SiC^foam^ at 60 °C is conventionally assumed equal to 1. Reaction conditions: [H_2_S] = 1 vol.%, [O_2_] = 2.5 vol.%, O_2_-to-H_2_S ratio = 2.5, [H_2_O] = 30 vol.%, balance helium, reaction temperature = 40, 60 and 80 °C, GHSV (STP) = 1200 h^−1^.

The temperature of the gas effluent can vary depending on its source and it can dramatically affect the catalyst performance in the process. Some state-of-the-art desulfurization catalysts are highly sensitive to the temperature of the H_2_S-containing effluent and control of the inlet gas temperature is often required to optimize their performance. Sun et al. [11] reported on the use of N-rich mesoporous carbons for H_2_S conversion in discontinuous mode at low temperatures, showing a significant decrease in the catalyst performance while increasing the reactor temperature in the 15–80 °C range. Therefore, the development of catalytic materials for the desulfurization process that are able to operate efficiently and selectively in a relatively wide range of temperatures is a highly challenging and desirable goal. Hereafter, we investigated the influence of the gas inlet temperature (40, 60 and 80 °C) on the desulfurization performance of NiS_2_/SiC^foam^. Figure 8 illustrates the catalyst performance (H_2_S conversion) as a function of the relative catalysts breakthrough times at the different temperature values.

To this aim, the breakthrough time (when H_2_S conversion starts to decrease) for NiS_2_/SiC^foam^ at 60 °C is conventionally assumed to be equal to 1. It can be inferred that H_2_S conversion increases while increasing the reaction temperature within the selected range of values. Indeed, the relative catalyst breakthrough time increases from 0.8 (at an inlet gas temperature of 40 °C) to 1.5 (at an inlet gas temperature of 80 °C). Such results highlight the ability of the NiS_2_/SiC^foam^ catalyst to effectively operate in a discontinuous mode within a relatively large range of temperatures for highly H_2_S enriched effluents without the need of enrichment units.

As expected, an increase in the reaction temperature translates into a decrease in the sulfur selectivity. In line with the literature precedents [49], the higher the desulfurization temperature the higher the H_2_S over-oxidation to SO_2_. Therefore, sulfur selectivity on NiS_2_/SiC^foam^ decreases from 99.6% at 40 °C down to 96% at 80 °C (see Figure S5 of the Supplementary Material).

## 4. Conclusions

This work describes the synthesis and use of a NiS_2_-decorated SiC foam as a highly efficient and selective low-temperature desulfurization catalyst with unprecedented sulfur-storage capacity (up to 140 wt.%) in the discontinuous treatment of highly H_2_S enriched (from 0.5 to 1 vol.%) gas effluents. The remarkable desulfurization performance of NiS_2_/SiC^foam^ can be attributed to the unique chemico-physical and morphological properties of the SiC foam that allow good reagents accessibility to the catalyst active sites, even when large deposits of sulfur are formed. In particular, the dual hydrophobic/hydrophilic character of the SiC support, due to the presence of a localized amorphous SiO_2_/SiO_x_C_y_ phase, creates preferential areas for the anchoring of the catalyst active phase and for the growth of sulfur deposits, respectively. This amphiphilic surface characteristic also provides a rationale for the sulfur agglomeration path.

In addition, NiS_2_/SiC^foam^ presents long-term stability in the desulfurization of gas effluents containing H_2_S concentrations as high as 1 vol.% with no apparent alteration in its performance after several catalytic/regeneration cycles. Therefore, it represents an excellent candidate to be employed in discontinuous desulfurization processes (i.e., Sulfreen, Doxosulfreen) as well as in the treatment of sour gas at low H_2_S concentrations (i.e., from 0.5 to 4 vol.%), where the traditional combined Claus/Super-Claus process is unable to operate.

## Supplementary Materials

Supplementary Materials are available online.
