# Supplementary material for: Nickel Sulfides Decorated SiC Foam for the Low Temperature Conversion of H2S into Elemental Sulfur
Source: Molecules. 2018 Jun 25;23(7):1528. doi: 10.3390/molecules23071528 (PMC6099843; doi:10.3390/molecules23071528)
Supplement: Supplementary file 1 [file molecules-23-01528-s001.pdf]

Supplementary material

Nickel Sulfides Decorated SiC-foam for the Low Temperature Conversion of H_2S into Elemental Sulfur

Cuong Duong-Viet,[1,2,*] Lam Nguyen-Dinh,[3] Yuefeng Liu,[1,4,*] Giulia Tuci,[5] Giuliano Giambastiani [1,5,6,*] and Cuong Pham-Huu,[1,*]

[1] Institute of Chemistry and Processes for Energy, Environment and Health (ICPEES), UMR 7515 CNRS-University of Strasbourg (UdS), 25, rue Becquerel, 67087 Strasbourg Cedex 02, France; cuong.pham-huu@unistra.fr (C. P.-H.)

[2] Ha-Noi University of Mining and Geology, 18 Pho Vien, Duc Thang, Bac Tu Liem, Ha-Noi, Vietnam; duongvietcuong@humg.edu.vn (C. D.-V.)

[3] The University of Da-Nang, University of Science and Technology, 54, Nguyen Luong Bang, Da-Nang, VietNam

[4] Dalian National Laboratory for Clean Energy (DNL), Dalian Institute of Chemical Physics, Chinese Academy of Science, 457 Zhongshan Road, 116023 Dalian, China; yuefeng.liu@dicp.ac.cn (Y. L.)

[5] Institute of Chemistry of OrganoMetallic Compounds, ICCOM-CNR Via Madonna del Piano, 10 – 50019, Sesto F.no, Florence, Italy; giuliano.giambastiani@iccom.cnr.it (G. G.)

[6] Kazan Federal University, 420008 Kazan, Russian Federation

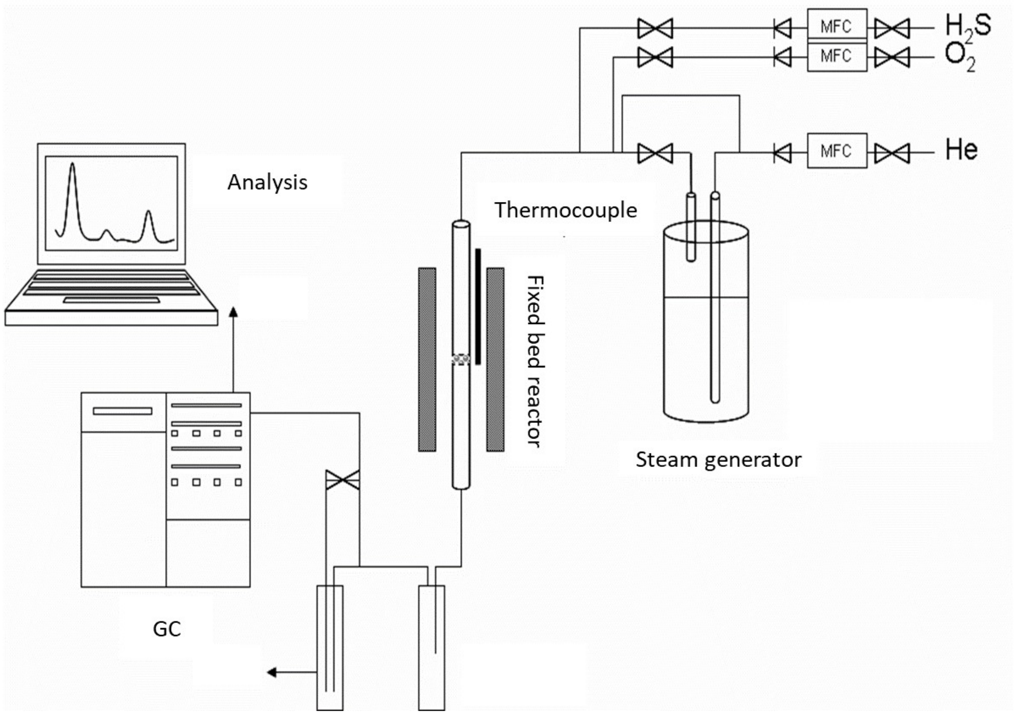

Figure S1. Schematic illustration of a desulfurization apparatus for testing NiS_2-decorated SiC samples

Table S1. Specific surface area (SSA) and pore size distribution of pristine SiC^{foam} and its composites. SSA values and total pore volume of SiCring and SiCextr have been also reported for the sake of completeness.

	SSA[a] (m²/g)	Total pore volume (cm³/g)[b]	Average pore size (nm)
SiC^{foam}	30	0.17	25
NiO/SiC^{foam}	35	0.12	18
NiS_2/SiC^{foam}	31	0.13	16
SiC^{ring}	26	0.14	23

SiC^{extr}	25	0.17	26

[a] Brunauer-Emmett-Teller (BET) specific surface area (SSA) measured at T = 77 K. [b]Total pore volume determined by using the adsorption branch of N_2 isotherm at p/p_0 = 0.98.

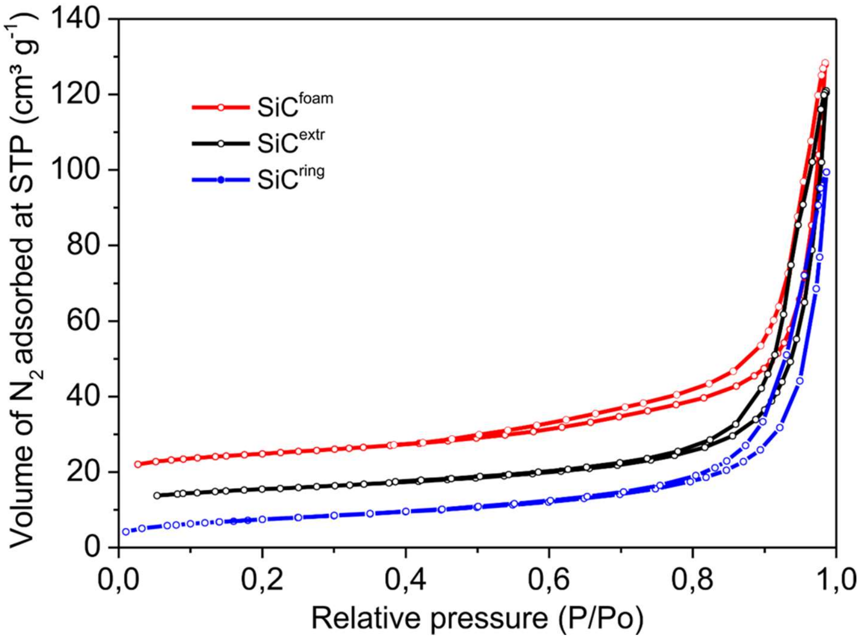

Figure S2. Nitrogen adsorption−desorption isotherm linear plots (BET) of **SiC^{foam}** (-○-), **SiC^{extr}** (-○-) and **SiC^{ring}** (-○-) recorded at 77 K.

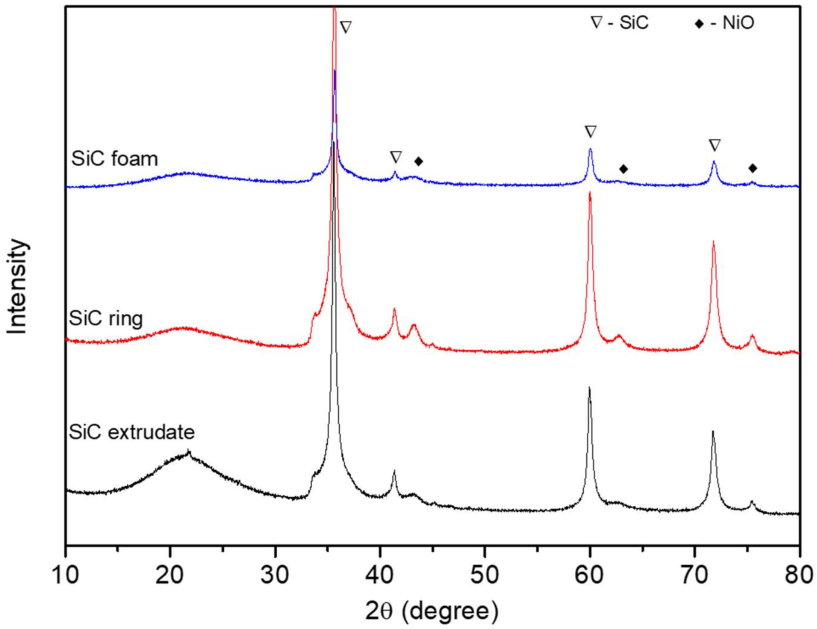

Figure S3. PXRD analysis of freshly prepared NiO on SiC^{foam}, SiC^{extr} and SiC^{ring}.

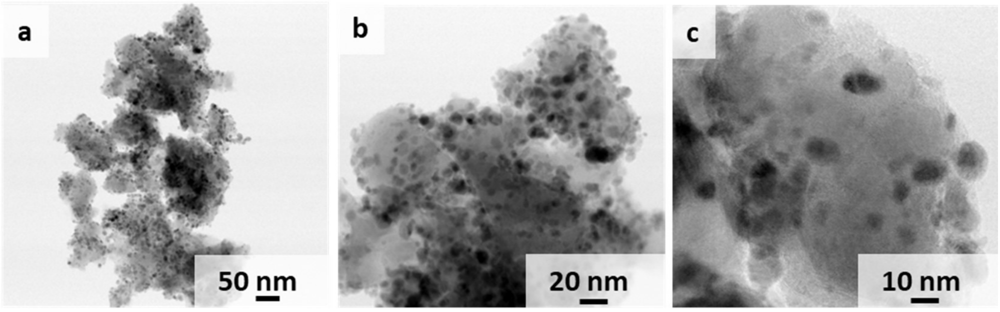

Figure S4. Representative TEM micrographs of NiO nanoparticles prepared following the procedure outlined in the manuscript for SiC supports decoration. A finely powder SiC support has been selected for the preparation of Ni-based composites to be employed for TEM characterization analysis. All images show a relatively well dispersed NiO NPs with size distribution between 5 and 8 nm.

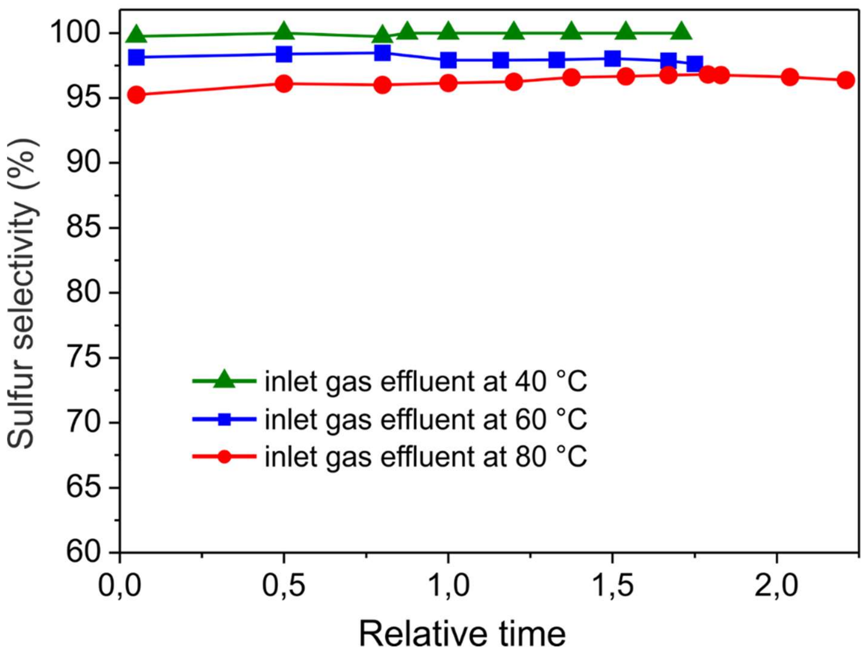

Figure S5. Sulfur selectivity on NiS_2/SiC^{foam} catalyst in the desulfurization process at variable temperatures. Reaction conditions: $[H_2S]$ = 1 vol.%, $[O_2]$ = 2.5 vol.%, O_2-to-H_2S ratio = 2.5, $[H_2O]$ = 30 vol.%, balance helium, reaction temperature = 40, 60 and 80°C, GHSV (STP) = 1200 h^{-1}.
